# Science and Islamic ethics: Navigating artificial womb technology through Quranic principles

**DOI:** 10.1016/j.heliyon.2024.e36793

**Published:** 2024-08-23

**Authors:** Muhammad Ahmad Ibrahim AlJahsh

**Affiliations:** College of Sharia and Islamic Studies, Al-Qasimia University, Sharjah, United Arab Emirates

**Keywords:** Science and religion, Artificial womb technology, Reproductive technologies, Quranic ethics, Bioethics

## Abstract

This paper explores the complex interplay between scientific innovation and religious ethics, with a specific focus on the ethical implications of Artificial Womb Technology (AWT) as interpreted through the lens of Quranic teachings on the essence of life. The objective is to meld the burgeoning field of reproductive technologies with the foundational principles of Islamic theology through an examination of Islamic jurisprudential rulings, contemporary bioethical discourse and innovations in reproductive technology. In addition to attention given to the compatibility of AWT with Islamic teachings concerning the sanctity of life, there is also a focus on the concept of motherhood and the preservation of family structure. This study undertakes an extensive exploration of both historical and contemporary interpretations of Islamic precepts, culminating in the establishment of an ethical framework. This framework is designed to harmonise religious doctrines with the exigencies of reproductive science by proposing normative guidelines for the ethical implementation of AWT and similar technologies. This paper makes a substantial contribution to academic discourse on science and religion by integrating advancements in reproductive health technology with moral principles intrinsic to the Islamic faith.

## 1 Introduction

This paper is situated at the confluence of evolving reproductive technologies and the venerable tenets of Islamic theology. Its central aim is to scrutinise the ethical contours of complete or full ectogenesis, or Artificial Womb Technology (AWT), through the interpretive lens of Quranic edicts pertinent to the sanctity of life and procreation, thereby illustrating the potential for harmonisation between this technology and Islamic ethics.

Artificial Womb Technology (AWT) represents a ground-breaking advancement in reproductive and neonatal care, aiming to support the growth and development of foetus outside the human body. AWT facilitates gestation ex utero, creating a unique human entity termed a "gestateling," distinct from a foetus or a newborn preterm neonate [33]. AWT involve creating an artificial environment that mimics the conditions of the human uterus [55]. This typically includes a sealed, fluid-filled chamber that houses the foetus, connected to an oxygenator that functions like a placenta to provide oxygen and remove waste. Nutrients and hormones are delivered through the fluid, while sensors monitor vital signs and adjust conditions as needed [63]. This technology has the potential to significantly improve outcomes for preterm infants and reshape the landscape of reproductive rights and ethics.

This analysis is significant for affirming the compatibility of technological innovations with the moral imperatives enshrined in religious doctrine, while also illuminating the moral and theological considerations that AWT introduces within the Islamic ethical landscape. Through engaging in a thorough examination of the Islamic responses to the ethical complexities introduced by AWT, this study endeavours to construct an ethical framework that can navigate the subtle interplay between the advancement of AWT and the Islamic bioethical of literature. This endeavour aims to foster a comprehensive and enlightened dialogue among the scientific and religious communities, thereby enriching the collective ethical discourse and understanding within this evolving domain.

This study examines the ethical complexities of Artificial Womb Technologies through Islamic bioethical principles. It integrates contemporary discourse and classical Islamic scholarship to provide a thorough analysis. The research investigates the application of established Islamic ethical tenets in formulating an initial framework for evaluating AWT, recognising the unprecedented challenges this technology presents. Given the evolving nature of both technological progress and Islamic Jurisprudence, this work identifies areas necessitating further theological and biological deliberation as AWT advances. The study's objectives contribute to ongoing discussions about AWT's ethical implications in Islamic contexts and establish a foundation for further research in this field. This approach offers insights into how Islamic principles might guide the ethical implementation of AWT, balancing scientific advancement with religious and moral imperatives.

Within Islamic bioethics, there exists a profound and multifaceted dialogue regarding the inception of human life and its attendant ethical implications. This academic conversation is characterised by divergent viewpoints. For instance, the assertion that life commences at the moment of conception [1] stands in stark contrast to the view that ensoulment occurs at a subsequent gestational phase [2]. While the former perspective views embryogenesis as a continuous trajectory, suggesting that the presence of a complete genetic code at conception heralds the emergence of life [3,4], the latter stance hinges on certain embryonic developments, specifically the initiation of neural activity which is viewed as the quintessential indicator of life [5], resonating with the Islamic notion of ‘soul-breathing’. This study traces the discourse surrounding the inception of life and related issues by conducting an in-depth analysis of *Quranic* interpretations, insights from early Islamic luminaries and contemporary scientific research. The amalgamation of theological doctrines, legal reasoning and scientific advancements situates this paper within the tradition of Ijtihad, which underscores the necessity for continuous re-evaluation and discourse in light of emerging scientific insights and established theological doctrines [2]. This approach to Islamic bioethical discussions is predicated on the collaborative endeavours of religious scholars and medical practitioners who are committed to reconciling theological and scientific perspectives. Their ongoing dialogue and the spirit of collaborative *Ijtihad* exemplify an attempt to craft a versatile and comprehensive approach to Islamic bioethics that is responsive to scientific advancements while remaining firmly anchored in theological understanding.

The seminal contributions of Muhsin, Chin and Padela on the subject of Artificial Womb Technology (AWT) within the context of Islamic jurisprudence [6] provide an essential ethico-legal perspective for navigating the landscape of AWT through the prism of Islamic legal maxims. Their pivotal work argues for the conditional acceptance of AWT, particularly when it aligns with the higher objectives of Shariah, such as the preservation of life and the advancement of medical practices, while also delineating its impermissibility in scenarios that deviate from these core principles. By building upon their insights and incorporating other Islamic viewpoints, including detailed Quranic interpretations, the current analysis seeks to furnish more understanding of the ethical dimensions of AWT within Islamic jurisprudence.

The contributions of Romanis are incisive in this field. Romanis explore the ethical and scientific intricacies of AWT [7,8], positioning it as a ground-breaking innovation in healthcare and a critical area for medical research. This technology is portrayed as an innovation beyond conventional neonatal care, with the potential to significantly improve extracorporeal gestation, thus reducing mortality and morbidity rates in premature infants. Romanis’ analysis, which includes an examination of the biobag and EVE platform, highlights the array of promising possibilities and inherent challenges of AWT. Moreover, it emphasises the ethical uncertainties and risks associated with classifying AWT as a novel clinical application, advocating for its designation as medical research that still requires thorough ethical and clinical due diligence before human application.

Ghaly, another pioneering researcher in the field, offers a critical examination of AWT's ethical and legal contours within the Islamic bioethical framework [2], discussing the ethical dilemmas surrounding the categorisation of AWT as either medical research or an innovative therapeutic intervention, particularly concerning preterm infants. Ghaly's comprehensive exploration of AWT's ethical implications contributes significantly to the dialogue on integrating cutting-edge reproductive technologies with Islamic ethical considerations.

In a distinct vein, Inhorn's seminal works [9,10], explore the sociocultural and ethical landscapes of IVF in Muslim societies, focusing on Egypt. Her later study investigates personal narratives and broader social ramifications of IVF use, articulating the complex personal and communal narratives surrounding these technologies, whereas the earlier publication examines the broader cultural and religious contexts that influence the assimilation and modification of IVF practices. Together, they offer a detailed comparative and empirical analysis that illuminates how the interplay of religious beliefs, local cultural traditions and medical practices influences the acceptance and adoption of reproductive technologies. Inhorn's (2003) [9] ethnographic investigation comprised an extensive series of over 200 in-depth interviews with a diverse cohort of IVF patients, medical practitioners, and religious scholars in Egypt, conducted over a two-year period. This rigorous interview process was complemented by meticulous participant observation in IVF clinics, affording an understanding of the practical application of Islamic principles in a real-world assisted reproductive technology settings. This methodological approach facilitated a comprehensive examination of the intricate interplay between religious convictions, cultural norms, and medical practices in shaping attitudes towards and adoption of reproductive technologies within Islamic contexts.

The aim of this study is to construct an ethical framework grounded in a detailed analysis of Islamic jurisprudence and enriched by insights from *Hadith*, *Ijma’* (consensus) and *Qiyas* (analogical reasoning). This paper concludes by emphasising the necessity for ongoing dialogue and cooperation between Islamic and biomedical ethics to effectively navigate the complexities introduced by these technological advancements.

## 2 Quranic foundations: the sanctity of life and birth in Islamic ethics

The Quran articulates the inviolability of life and the divinely guided process of gestation, positioning them as pivotal tenets within Islamic ethics. The term *ḥaml*, representing pregnancy in these sacred texts, refers to the genesis of life beginning with the zygote's initial implantation within the uterus.

Within the Islamic scholarly tradition, ensoulment—the infusion of the soul into a nascent life form, endowing the entity with sacred inviolability and rights—is more commonly believed to occur at a decisive post-conception phase. This ensoulment period, interpreted variably as spanning between 40 and 120 days post-conception, establishes a critical ethical threshold after which any act of termination is stringently interdicted [5,[11], [12], [13]], due to the doctrine of the sanctity of life. This is rooted deeply in Quranic injunctions that vehemently denounce the pre-Islamic Arabian practice of infanticide; thus, it extends universally beyond the confines of a single religious identity. As implied by the concept of ensoulment, reproduction in Islam is envisioned not merely as a biological or societal act but as a divine covenant imbued with significant spiritual and familial responsibilities [14]. Through select verses, the Quran unveils the journey of life from its inception to birth, illustrating the intricate divine intelligence that underpins this process [15,16]. These narratives not only accentuate the sanctity and marvel of life but also underscore the ethical imperative towards its protection and dignity.

As mentioned in Quranic verses from Surah al-An'am- (the Cattle) (6:151) and Surah al-Isra’ (the Night Journey) (17:33), a paramount moral duty is ascribed to preserve life, with a categorical prohibition on unjust killing. For instance, while Islam generally prohibits abortion based on the principle of life's sanctity, some Muslim scholars allow for exceptions in cases where the mother's life is at risk or in cases of severe foetal abnormalities that are incompatible with life [25,42]. These rulings draw on the Islamic principles of the lesser of two evils (*akhaf al-dararayn*) and the prevention of harm (*mafsadah*) respectively. Such principles lay the foundation for Islamic bioethical considerations, guiding the ethical navigation across various medical-ethical quandaries, including the realms of abortion, euthanasia and end-of-life care [17], and they are of cardinal importance within the context of Islamic discourse on ectogenesis or AWT. This study endeavours to critically engage with Quranic teachings to address the emergent ethical challenges posed by AWT, such as the moral status of embryos in artificial environments, the ethical prerequisites for AWT's permissibility and its potential implications on familial dynamics and societal norms.

Islamic jurists and bioethicists engaged in an appraisal of AWT's admissibility weigh its potential merits in preserving the lives of premature neonates and aiding infertile couples against its congruence with Islamic tenets on life's sanctity, lineage preservation and the natural order [18]. Scholarly discourse on AWT frequently deliberates whether the artificial gestational milieu impacts the traditional understanding of ensoulment and its temporal markers. Some scholars contend that ensoulment, as a divine decree, remains impervious to the gestational mode, thereby positing that AWT's utilisation in sustaining foetal life does not contravene Islamic doctrinal teachings [19]. Conversely, others voice concerns over the potential disruption to the natural gestational process and its spiritual dimensions, advocating for prudence and further scholarly inquiry [20]. In navigating these intricate considerations, Muslim scholars draw upon the rich corpus of Islamic jurisprudence, employing *ijtihād* (independent reasoning), *qiyās* (analogical deduction) and *maṣlahah* (consideration of public interest) to forge sophisticated verdicts that harmonise universally recognized Islamic ethical values with medical advancements [21].

### 2.1 The Quranic exegesis of Life's genesis from conception to birth

The Quran depicts the journey from conception to birth a divinely guided sequence. Scripture presents a vivid chronological account of human development, commencing with the zygote's inception and culminating in the emergence of a fully formed infant. This primarily draws upon verses from Surah *Al-Mu'minun-*“the Believers”(23:12–14), and Surah Al-Hajj- “the Pilgrimage” (22:5), which describe the stages of embryonic growth from a drop of fluid to a complete human being.

Surah Al-Mu'minun states: "And certainly did We create man from an extract of clay. Then We placed him as a sperm-drop in a firm lodging. Then We made the sperm-drop into a clinging clot, and We made the clot into a lump [of flesh], and We made [from] the lump, bones, and We covered the bones with flesh; then We developed him into another creation. So blessed is Allah, the best of creators" (Quran 23:12–14).

This gradual progression not only depicts the physical stages of embryological development but also illuminates the divine artistry and complexity imbued in the creation of life [22].

Surah Al-Hajj adds: "O people, if you should be in doubt about the Resurrection, then [consider that] indeed, We created you from dust, then from a sperm-drop, then from a clinging clot, and then from a lump, formed and unformed, that We may show you. And We settle in the wombs whom We will for a specified term, then We bring you out as a child, and then [We develop you] that you may reach your [time of] maturity" (Quran 22:5).

These verses not only illustrate the physical stages of embryonic development but also highlight the divine hand in the creation of life.

Both of verses not only chronicle the physical progression of embryonic development but also underscore the precise timing of gestation, as decreed by divine will, culminating in the birth of a child who matures to full adulthood [22].

Far from merely providing a biological account, these Quranic verses also imbue each phase of growth with profound spiritual significance. The Islamic interpretation of embryology thus encompasses both the tangible and intangible realms, perceiving each stage as a manifestation of God's wisdom and power [23]. This holistic perspective significantly shapes the Islamic ethical stance on conception, embryonic development and birth, offering a comprehensive framework for moral and legal contemplation [2].

The Quran's portrayal of life's inception is central to Islamic bioethics, and informs Islamic legal rulings concerning the status the embryo and foetus [24] and fosters an appreciation for both the scientific understanding of embryology and its spiritual and ethical dimensions. Through the lens of Quranic teachings, Muslims scholars engage in an in-depth dialogue between religious doctrine and contemporary scientific advancements, ensuring a harmonious reverence for life alongside the pursuit of knowledge [25].

The Quranic discourse on the journey from conception to birth thus not only lays the theological groundwork for understanding human creation, but it also establishes an ethical framework for guiding adherents in their engagement with modern medical technologies [5].

### 2.2 Ethical, legal and moral dimensions of the sanctity of life in islam

In Islam, the significance of life's sanctity extends beyond abstract theological principles, affecting laws and ethics that guide individuals and communities. At the heart of the Quranic narrative on life's sanctity is *Sūrat al-Anʿām,* the Cattle, (6:151), And do not kill the soul which Allah has forbidden, except by right. This has He instructed you that you may use reason.' This verse unequivocally forbids the taking of a soul without just cause. This edict stands as a testament to the high regard Islam holds for life. The verse, interpreted by Islamic jurists and ethicists as a cardinal ethical principle, advocates for the preservation and protection of life. It establishes the conviction that life is inherently sacred, and its unjust taking is tantamount to the destruction of humanity in its entirety, as stated in 5:32, which reads: ‘Whoever kills a soul unless for a soul or for corruption [done] in the land – it is as if he had slain mankind entirely. And whoever saves one – it is as if he had saved mankind entirely’. Sūrat *al-Isrā’*, the Night Journey, (17:33), states ‘And do not kill the soul which Allah has forbidden, except by right’, reinforces this moral imperative to preserve life, asserting that even in the context of lawful retribution or capital punishment, adherence to the limits ordained by divine law is mandatory, and playing a essential role in shaping Islamic jurisprudence related to the permissibility of taking a life, mandating strict adherence to ethical and legal procedures in such grave circumstances.

The above verses are foundational in shaping Islamic ethics concerning issues ranging from abortion and euthanasia to the death penalty and warfare. Scholars such as Sachedina and Esposito have explored how these principles have been interpreted and applied in various situations, illustrating the adaptive and context-sensitive nature of Islamic jurisprudence [5,26]. For, while the sanctity of human life remains a paramount value in Islamic ethics, its application varies across different Islamic schools of thought and cultural contexts. This is manifested in the range of ethical directives, legal stipulations and moral reasoning prevalent in Muslim-majority societies on various medical issues [21], particularly those concerning the beginning and end of life such as abortion, the discontinuation of life support and organ donation and those that have been influenced by modern medical breakthroughs [27]. What the various rulings and arguments have in common is a deep-seated reverence for life, yet one that is reasoned and evidence-based [18].

While this analysis centers on Islamic ethical principles, many of the foundational values-such as the sanctity of life, human dignity, and justice-are shared across religious traditions. The Qur'anic injunction against unjustly taking life (Q.5:32) resonates with similar Biblical teachings [60]. Judeo-Christian and Islamic thought also share a reverence for human life as a sacred gift endowed by God [61]. These common principles highlight the universal nature of certain moral considerations in reproductive ethics, particularly as they pertain to emerging technologies like AWT [61].

### 2.3 Islamic Jurisprudence and the ethical discourse on birth

Within the rich tapestry of Islamic jurisprudence and rooted in the revered texts and traditions, there exists a comprehensive framework for grappling with the ethical implications of birth and life. Islamic scholars engage extensively with the Quran and *Hadith* to extract principles and develop rulings that inform ethical decision-making against the backdrop of contemporary medical advancements. A significant element in Islamic bioethics is the aforementioned concept of ensoulment. While interpreted variably, it is most often understood to occur approximately 120 days after conception. This threshold, which is derived from *Hadith* interpretations [2], represents a significant milestone in foetal development and bears considerable weight in the Islamic discourse on abortion and the utilisation of assisted reproductive technologies. It marks a moral boundary, guiding permissible interventions in prenatal care and broader medical practices [22]. As advancements in medicine increasingly allow for earlier and more complex interventions, Islamic scholars must consistently re-evaluate these teachings, balancing the sanctity of life with the broader concern for personal well-being.

The ethical discourse in Islam also rigorously examines the intricacies of in vitro fertilization (IVF) and related technologies. While generally accepted under specific conditions– such as upholding lineage and marital bonds– these modalities are critically analysed to ensure alignment with the overarching principles of Islam [28]. This careful examination reflects the Islamic emphasis on the integrity of the family unit and the welfare of the child, ensuring that technological progress serves the collective good and adheres to the ethical and social framework of the community.

Extending beyond mere technical considerations, Islamic ethical thought offers a holistic perspective on the individual and societal aspects of birth. It encompasses the rights and responsibilities of parents, the welfare of the child and the broader societal ramifications of reproductive decisions. The bioethical discourse explores issues of justice, equity and the implications for future generations, advocating for a comprehensive decision-making process that considers the dignity of individuals, community welfare and resource stewardship [29]. In this process, Islamic jurists employ *Ijtihad* to understand how religious texts and principles can be applied to new contexts. This dynamic and reflective approach ensures that Islamic bioethical guidance is both relevant and adaptable, addressing the evolving capabilities of the medical field while remaining firmly anchored in the Quran and Sunnah [30].

In summary, Islamic jurisprudence offers an extensive ethical framework for navigating the complexities associated with birth and reproductive technologies by continuously engaging sacred scripture, the ethical principles inherent within scripture and emerging medical issues. In this way, Islamic scholars provide a robust framework for decision-making that honours life's sanctity, promotes justice and fosters the well-being of individuals and communities. This enduring scholarly pursuit reflects a deep commitment within Islam to maintaining ethical integrity and embracing the opportunities and challenges of contemporary medicine.

## 3 The ethical implications of AWT

### 3.1 Primary concerns

The advent of partial ectogenesis through AWT has heralded a host of ethical considerations that demand close scrutiny. As a pioneering venture into medical technology, AWT's experimental stages evoke profound ethical dilemmas, especially concerning the use of foetuses in the later stages of development. Primary considerations include ensuring informed consent, rigorously evaluating the risk-benefit ratio and clarifying the ethical and legal status of developing human entities during initial research phases.

In addition, the rise of ectogenesis is set to dramatically shift established discourses on abortion and fetal rights, impacting discussions on foetal viability and ethical debates concerning the right to life. It can thus also be expected to influence public discourse and shape legal frameworks. Such developments call for a comprehensive and systematic ethical assessment [31]. The ability to sustain embryos and foetuses externally could lead to the commodification of gestation, turning a natural biological process into a commercialised service and raising profound ethical issues pertaining to the essence of parenthood and the risk of exploitation or disparate access to the technology. The commodification of pregnancy through AWT would be problematic from an Islamic perspective, as it reduces the sacred act of childbearing to a mere commercial transaction. Islam emphasises the importance of familial bonds and the nurturing role of mothers, which could be undermined by the commercialization of gestation [58,59]. For instance, the use of AWT for non-medical purposes, such as convenience or career advancement, would be considered a violation of the Islamic principle of preserving the natural order (*fitrah*) and the sanctity of the womb [20]. Such use could lead to the exploitation of women from disadvantaged backgrounds, who may be pressured to “rent out” their wombs for financial gain, a practice that is prohibited in Islam [57,59]The implementation of AWT signifies substantial shifts in traditional parenting paradigms and family structures that have broad implications for society as a whole. On the immediate level, AWT necessitates redefining the legal construct of parentage and re-evaluating the associated responsibilities and rights of parents.

The impact of AWT on abortion laws is another crucial consideration from an Islamic perspective. While Islam generally prohibits abortion after ensoulment (120 days post-conception), the permissibility of terminating a pregnancy within an artificial womb remains a matter of debate among Islamic scholars [2,18]. Some may argue that the absence of physical and emotional burdens on the mother could make the termination of an ectogenetic pregnancy more difficult to justify, as the principle of the lesser of two evils (*akhaf al-dararayn*) may not apply [42]. Others may contend that the principles of life's sanctity and the prevention of harm (*mafsadah*) would still apply, necessitating a case-by-case evaluation of the ethical permissibility of termination, taking into account factors such as foetal viability and the presence of severe abnormalities [18]. Like many advanced medical technologies, AWT's availability might be unequal, potentially exacerbating socio-economic divides. Ethical considerations must address the distribution of this technology by questioning who has access and the wider implications for marginalised groups. Careful reflection on justice, resource allocation, and the potential ramifications on disadvantaged populations is needed to protect individual rights.

Despite these ethical complexities, the potential benefits of AWT are significant and warrant recognition. AWT offers possibilities for individuals unable to engage in conventional pregnancy, mitigates associated gestational and childbirth risks, and improves survival rates and health outcomes for preterm infants. Therefore, ethical discussions should weigh these substantial advantages against potential risks. As AWT progresses, maintaining an ongoing, adaptable ethical dialogue is important to ensure that its development and deployment adhere to safety, equity and communal welfare principles [32].

An essential first step is distinguishing AWT from traditional neonatal intensive care practices to establish specific ethical guidelines and regulatory frameworks. The next step involves considering the challenges associated with children born through AWT. The progression of AWT invites a reconsideration and possible redefinition of ‘birth’.

Romanis [7] has proposed the term ‘gestateling’ to refer to the subject of partial ectogenesis, as these infant, though expelled from the womb, still possess fetal physiology and have not yet taken their first breath, which is legal requirement for ‘birth’ in some jurisdictions [34,56]. This new terminology is vital for a legal and ethical differentiation, providing distinct nomenclature for these individuals [7]. However it is important to note that the classification of AWT subjects as ‘gestatelings’ is not universally accepted, and Islamic bioethical frameworks may offer unique insight into the moral status of these entities. Distinct ethical challenges may emerge due to differences between ‘gestatelings’ and traditionally born infants, such as disparities in cognitive functions or health outcomes, and these challenges need to be addressed. Those ‘birthed’ through AWT deserve to have their unique conditions and futures acknowledged prior to the implementation of the technology.

From an Islamic perspective, the concept of ensoulment [2] could serve as a significant marker for determining the moral status of the 'gestateling.' If ensoulment has occurred, the 'gestateling' may be considered a person deserving of full moral rights and protections, regardless of their location inside or outside the womb [22].

Determining the legal status and rights of gestatelings is a crucial aspect of AWT's ethical discourse. Society must provide these individuals with rights and protections equal to those afforded to traditionally born infants, including legal recognition and entitlements. The progression of AWT invites a reconsideration and possible redefinition of 'birth.' While any potential adverse outcomes in partial ectogenesis are an intrinsic risk of prematurity, animal studies suggest that preterm animals “born” via AWT have better outcomes compared to those receiving traditional care [33]. However, it is essential to avoid comparing the outcomes of preterm infants, whether 'born' via AWT or not, with those of term infants, as prematurity itself poses significant health challenges [34]. Ultimately, creating a comprehensive ethical framework that addresses these and other ethical considerations is essential. Such a framework should guide medical and scientific professionals as well as policymakers, ensuring AWT's deployment is consistent with principles of respect, dignity and justice. This necessitates an enduring ethical commitment to the technology, namely, one that adapts to new insights and challenges as they emerge.

### 3.2 Deriving ethical guidelines from quranic teachings

For Muslims, an ethical framework for the oversight of AWT will be based on Islamic principles and values found in the Quran. It will thus emphasise humanity's role as stewards or vicegerents of Allah on Earth. Indeed, the Islamic ethical concept of *Khilafah* (stewardship) is informed by various Quranic verses which, while not directly addressing AWT, are applicable to ethical considerations in contemporary technology including those that significantly impact the survival and health of premature infants [33,33,35]. These teachings are summarised below.

• Stewardship: Quran 2:30 (Surah *Al*-*Baqarah*-the Cow) discusses the role of humans as stewards on Earth, emphasising the responsibility entrusted to humanity to uphold the world's order, including the ethics governing technology and medicine.
• Sanctity and Preservation of Life: Quran 5:32, Surah *Al-Ma'idah* (the Table) emphasises the immense value of each life, positing that saving one life is akin to saving all of humanity. This verse is a cornerstone of Islamic bioethics that stresses the importance of life-preserving technologies, including those supporting premature infants.

Several guidelines for the ethical development and deployment of contemporary technologies such as AWT can be derived from this scriptural foundation. They include, first, that the AWT technology respect natural biological processes and uphold the sanctity of life. The research and application of such technology should proceed with ethical diligence and integrity, adhering to the moral guidelines outlined in Islamic jurisprudence. A thorough evaluation of AWT's impact on the foetus or gestateling must be conducted since the Quran emphasises the intrinsic value of life from its inception, imposing an ethical responsibility on technologies that interact with human life. In the context of artificial wombs, this translates into a commitment to the care and protection of the foetus or gestateling, ensuring the respect and sanctity of the life it supports. Second, equitable access to AWT technology must be ensured to reflect the Islamic principles of justice and fairness. The development and application of AWT must ensure that individuals from diverse socio-economic backgrounds can benefit from this innovative technology. Third, since the Quran assigns humans the responsibility of environmental stewardship, compelling them to protect and preserve the Earth, the development and application of AWT must consider its ecological implications. This commitment to environmental stewardship is an integral part of ethically responsible technological innovation. Fourth, dialogue on this topic must include the perspectives of scientists, ethicists, theologians and the broader community. Islam places a high value on the acquisition and dissemination of knowledge; hence, a commitment to transparency and informed dialogue is crucial to understanding the ethical, societal and medical implications of AWT and navigating the complexities associated with it.

Ultimately, these principles provide a basic framework for addressing the ethical challenges posed by AWT and ensuring that its development and application are consistent with the fundamental moral and ethical objectives of Islamic teachings.

## 4 Formulating an Islamic ethical paradigm for reproductive technology

### 4.1 An interdisciplinary approach

The formulation of Islamic ethical guidelines for reproductive technologies requires an interdisciplinary approach that integrates insights from Islamic jurisprudence, bioethics and medical science.

The Quran underscores the sanctity of life, as exemplified in the verse ‘And do not kill the soul which Allah has forbidden, except by right’ (Quran, 17:33), while the *Hadith* literature, including sayings of the Prophet Muhammad (peace be upon him), emphasising the importance of seeking medical treatment: ‘Allah has not created a disease except that He also has created its treatment’ (*Sahih al-Bukhari*, Book 76, Hadith 1). These scriptural foundations affirm the permissibility of employing medical advancements such as AWT to preserve life (*hifz al-nafs*) and intellect (*hifz al-'aql*) [36] and to alleviate suffering. However, the utilisation of AWT must also be guided by other ethical principles that are foundational to Islam, including the preservation of lineage (*ḥifz al-nasl*), recognized as one of the five essential objectives (*maqāṣid*) of *Shari'ah* [36]. Additionally, it must consider the importance of family integrity, adherence to the principle of non-maleficence *(la ḍarar wa la ḍirar*), and the imperative of informed consent, as emphasised by contemporary Muslim jurists. When addressing the complex nature of AWT, it is vital to consider various issues, including the ethical implications of embryo selection and the management of surplus embryos. As stated earlier, the Quran (23:14) alludes to the stages of human development in the womb, providing a basis for discussing the moral status of the embryo at different stages of development; however, it does not settle the issue of utilising surplus embryos for research purposes. Some Muslim scholars [18], argue that embryos in the early stages of development may be used for research aimed at advancing medical knowledge and treatments, provided that strict ethical guidelines are followed, whereas others adopt a more cautious stance [5], depending on their understanding of when life begins.

Addressing the ethical implications of AWT involves examining these and other issues, including embryo selection and the use of donor gametes. Various Quranic passages related to human gestation (e.g. Quran 19:22, 31:14 and 46:15) have been utilised to provide a scriptural underpinning that can guide embryonic stem cell research (ESC). Based on this, the Islamic Fiqh Council has permitted the use of surplus embryos from IVF for stem cell research [37], provided they are not created solely for this purpose and are used before 120 days post-conception. This decision, based on the Quran and Hadith, reflects a meticulous Islamic jurisprudential approach that weighs the potential benefits of medical research against the respect for early human life [23].

However, the use of IVF with donor gametes or surrogacy arrangements that involve third parties would be considered unacceptable in Islamic law, as they violate the principles of lineage preservation (*hifz al-nasl*) and the sanctity of the marital bond [10,19]. For example, using donor sperm in IVF would be prohibited, as it introduces the possibility of confusion regarding the child's paternity and disrupts the legal and social norms surrounding family formation in Islam.

### 4.2 Advantages of integrating islamic principles in reproductive technology practices

The integration of Islamic principles within the domain of reproductive technology offers several advantages. One is the structure that it brings to the ethical complexities of modern reproductive technologies. Grounded in the teachings of the Quran and Sunnah, the Islamic ethical framework provides clear directives for scientific progress that ensure reproductive technologies are employed responsibly, respectfully and with well-formulated objectives. Another advantage of this integration, and of paramount importance, is that it promotes ethical scientific inquiry. Given AWT's emergent status and its limited application to aiding extremely premature infants through partial ectogenesis, its use is currently being scrutinised through the lens of Islamic ethical imperatives aimed at preserving life. By firmly opposing the deliberate creation of pre-embryos for ESC research, whether through standard IVF or somatic cell nuclear transfer (SCNT), the incorporation of Islamic principles reflects a commitment to research practices that show reverence for the early stages of human life [38]. This aligns with a fundamental principle of medicine, which is to protect life and not endanger it. Recent ethical literature has reinvigorated discussions on the relation between AWT and the mandate to protect life by focusing on AWT as a method of neonatal care rather than the more remote possibility of complete or full ectogenesis [39]. Lastly, the incorporation of Islamic ethics into discussions of reproductive technologies has been beneficial to Islam itself in that it has prompted a more sophisticated discussion of when life begins.

## 5 Engaging Islamic ethics in the advancement of AWT

The discourse on reproductive technologies within Islamic scholarship is marked by an appreciation of these innovations’ potential to alleviate infertility challenges. However, it concurrently mandates a thorough ethical assessment of their broader implications. In addressing AWT, Islamic ethical inquiry proposes principles such as the sanctity of matrimonial bonds, the imperative of lineage clarity and the preservation of family structure, underscoring the paramount nature of child welfare and familial cohesion. Islamic researchers [29,40] play an essential role in the dynamic conversation as their contributions are instrumental in ensuring that the development and application of AWT align with the ethical, moral and societal tenets of Islam.

### 5.1 Enhancing ethical discourse on AWT within islamic bioethics

The Islamic ethical considerations surrounding surrogacy provide a useful framework for analysing AWT. Both technologies involve gestation outside of the mother's body, raising questions about the definition of motherhood and the rights of the resulting child. Just as many Islamic scholars prohibit surrogacy due to concerns about lineage confusion and commodification of reproduction [28,62], similar arguments may be applied to AWT, particularly in cases of full ectogenisis. However, the use of AWT for partial ectogenisis to save premature infants may be viewed more favourably under the Islamic principle of preserving life.

Rooted in Hadith literature, the Islamic concept of ensoulment is mentioned in the Bill of Health [41] and has been explored by contemporary scholars [6,42,43] who investigate its implications for bioethical considerations in the context of emerging technologies. The ethical inquiry into ensoulment within an artificially sustained gestational environment, as in the case of partial ectogenesispresents a complex debate among Islamic scholars. Some argue that ensoulment, being a metaphysical occurrence ordained by God, remains unaffected by the physical conditions of gestation, suggesting that AWT, as a life-support mechanism, does not inhibit the soul's endowment at the divinely appointed time. This view posits AWT, especially in aiding the viability of extremely premature neonates, as compatible with Islamic directives to protect life, especially insofar as it aids the viability of extremely premature neonates [7,8]. However, employing AWT before ensoulment necessitates thorough ethical scrutiny to maintain the delicate equilibrium between scientific progress and the spiritual and moral sanctity of life [44] and to ensure the technology's application does not breach Islamic principles regarding the ordained progression of human development [7,8].

### 5.2 Integrating AWT within the islamic ethical paradigm

Islamic tradition venerates the natural world as a manifestation of divine revelation and advocates for its profound study to enrich religious insight and expand the horizon of scholarly knowledge [45,46]. The Islamic approach views AWT and similar technologies with cautious optimism as potential instruments for preserving life and aiding those facing medical impediments to natural childbirth. However, the application of these technologies to bypass the natural gestation process entirely, as in the case of full ectogenesis may not resonate with Islamic ethical principles, It is important to note that full ectogenesis remains a speculative concept and has not yet been achieved [6]. Historically, the synthesis of Islamic scholarship and scientific inquiry, exemplified by the intellectual legacy of al-Ghazālī and contemporaneously interpreted by scholars like Afifi al-Akiti, has paved the way for the assimilation of scientific breakthroughs within an Islamic ethical context [1]. This synergy is notably significant in the realm of genetic counselling in assisted reproduction, where Islamic bioethical viewpoints harmonise scientific credibility with ethical integrity [47].

The plurality of interpretations within Islamic thought on biomedical progress mandates a flexible and inclusive approach in formulating an ethical framework, [48]. Ensuring that reproductive technologies such as AWT are congruent with Islamic ethical teachings and reflective of the socio-cultural dynamics of Muslim communities is significat for their ethical integration [49].

The Quran underscores the natural desire for offspring, acknowledging infertility as a significant trial and endorsing the pursuit of lawful remedies (Quran 14:46, 25:74, and 42:49–50). Islamic jurisprudence employs principles such as *Istihsan* (juristic preference) and *Sadd al-Dharai* (blocking the means) to navigate the ethical considerations surrounding AWT, emphasising the preservation of lineage and family integrity as per teachings of the Quran and Sunnah [50]. In this context, AWT might be deemed permissible for life-saving interventions in premature infants or to mitigate infertility challenges within the sanctity of marriage, upholding the foundational Islamic values of lineage preservation and marital fidelity. Conversely, employing AWT to circumvent natural childbirth in healthy women or to facilitate parenthood outside marital bounds may contravene Islamic ethical guidelines [44,51].

### 5.3 Key requirements of the present initiative

The initiative to integrate Islamic theological principles with scientific advancements in reproductive technology, specifically AWT, is a multifaceted endeavour that addresses the numerous ethical challenges posed by modern reproductive sciences. A key requirement of this initiative is to balance individual autonomy with established cultural norms, particularly in societies where reproductive technologies intersect with tradition. For instance, in regions like Egypt, the application of these technologies often requires respect for both individual choices and societal expectations [9]. The establishment of an ethical framework that respects personal rights while maintaining community values is therefore essential.

This endeavour also requires a thorough examination of religious doctrines, scientific breakthroughs and technological applications. On one hand, traditional cultural and religious views, including those pertaining to reproductive organs, which Islam considers sacred, must be considered. On the other hand, Islam's dynamic nature allows for the accommodation of societal changes. With rapid scientific advancements, there is a need to reassess and potentially reframe traditional Islamic views on motherhood, gestation and childbirth [10]. Modern reproductive technologies may alter long-established theological conceptions, necessitating a comprehensive reappraisal. A comparative analysis will draw parallels between Quranic injunctions against unjustly taking human life and contemporary Islamic perspectives on birth control and abortion, offering a refined view of Islamic bioethical stances on these pivotal reproductive matters [52]. Additionally, an examination of modern exegetical scholarship on gender-related themes in the Quran is needed to deepen the understanding of gender roles within Islamic theology [53]. The intellectual and scholarly contributions of renowned Islamic thinkers, such as Said Nursi, may be helpful in informing discussions about gender reform within Islamic discourse [54].

In summary, the proposed multi-faceted methodological approach to the incorporation of Islamic principles in reproductive technology aims to address the ethical challenges of these technologies in a holistic, synergistic and collaborative manner. In this way, it proposes to adapt Islamic principles to scientific progress while also contributing to the creation of ethically robust and culturally attuned reproductive technologies.

## 6 Conclusions and recommendations

This study has traversed the intricate nexus of Islamic theological tenets and contemporary reproductive technologies, with a focus on the ethical deliberations that AWT incites within the Islamic discourse on life and procreation. It brings to the forefront the indispensable role of Islamic bioethics in fostering child welfare and sustaining familial bonds amidst the challenges posed by medical advancements. The diversity of Islamic interpretations regarding the ethics of these advancements, reflective of the myriad cultures and individual viewpoints within the Muslim community, is duly recognized and respected.

At its core, the research advocates for a continued and collaborative dialogue between Islamic legal scholars and experts in reproductive technology to navigate the varied societal influences and necessities that inform reproductive choices. This partnership is crucial for crafting and refining a comprehensive framework for biomedical technology that is empirically and conceptually accountable, encompasses a wide array of reproductive technologies, and addresses broad issues such as embryo donation and genetic interventions. The aim of such a partnership is to unravel the ethical complexities that technologies such as AWT present within Islamic bioethics and to elucidate the ethical and legal conundrums associated with both gestational and traditional forms of surrogacy. Ultimately, the goal is to ensure that the adoption of reproductive technologies is congruent with the ethical, moral and socio-cultural fabric of the Muslim community.

The study champions a fluid interpretation of Islamic teachings, one that remains steadfast to the core principles of Islam while adaptively responding to the relentless march of biomedical technology. It posits that future ethical guidelines and discussions should unfailingly place child welfare and the sanctity of the family unit at the heart of bioethical considerations and technological applications. Moreover, the study calls for ongoing scholarly explorations that breach existing confines, to enrich the dialogue with a more diverse array of scholarly contributions and insights from across the broader of Islamic community. It advocates for a judicious and collaborative approach that venerates both religious doctrines and scientific knowledge, thereby paving the way for the ethical employment of reproductive technologies in a manner that preserves fundamental Islamic values pertaining to the sanctity of life and birth. This methodology is vital for the development of ethically sound and culturally sensitive reproductive technologies that can be seamlessly integrated with Islamic ethics.

## CRediT authorship contribution statement

**Muhammad Ahmad Ibrahim AlJahsh:** Writing – review & editing, Writing – original draft.

## Declaration of competing interest

The authors declare that they have no known competing financial interests or personal relationships that could have appeared to influence the work reported in this paper.
